# Medication-induced causes of delirium in patients with and without dementia: a systematic review of published neurology guidelines

**DOI:** 10.1007/s11096-024-01861-4

**Published:** 2025-02-19

**Authors:** Anita Elaine Weidmann, Guðný Björk Proppé, Rut Matthíasdóttir, Ivana Tadić, Pétur Sigurdur Gunnarsson, Freyja Jónsdóttir

**Affiliations:** 1 Institute of Pharmacy, Department of Clinical Pharmacy, Innsbruck University, Innrain 80, 6020 Innsbruck, Austria; 2 Faculty of Pharmaceutical Sciences, University of Iceland, Saemundargata 2; 102, Reykjavík, Iceland

**Keywords:** Clinical guidelines as topic, Delirium, Dementia, Medication therapy management, Neurology

## Abstract

**Background:**

While medication is a recognized risk factor of delirium, there is currently a lack of detailed information on managing and preventing medication-induced cases.

**Aim:**

This review summarizes the information provided in neurology guidelines on medication-induced delirium in patients with and without dementia to inform guidance on prevention and management strategies.

**Method:**

A systematic literature review was conducted across 114 neurological and medical organisations, Guideline Central and PubMed. Guidelines, consensus guidelines, white papers, frameworks, protocols, standard procedures, action plans and strategic documents detailing the prevention and management of medication-induced delirium in adults with or without dementia were included. Title and full-text screening was completed independently by two reviewers using PICOS. AGREE II was used to assess reporting quality. A data extraction tool was designed based on the Cochrane Effective Practice and Organization of Care Review Group (EPOC) checklist and a mixed methods approach to synthesis adopted. The systematic review protocol was registered with International Prospective Register of Systematic Reviews (PROSPERO) [ID: CRD42022366025].

**Results:**

Out of 143 guidelines identified, 30 were included. Information for 140 individual medications was extracted. Medications most frequently cited included sedatives (n = 24/80%), opioids (n = 22/73,3%), psychoactive drugs (n = 21/70%) + anti-convulsants (n = 14/46,7%), anti-cholinergic agents (n = 20/66,7%), antihistamines (n = 18/60%), and steroids (n = 16/53,3%). Despite a consistently high-quality rating (n = 19, 63,3%), the detail provided often lacks specificity about pharmacological mechanisms, individual risk, dosing instructions, associated symptoms, therapeutic alternatives and avoidable drug-drug combinations. In relation to dementia, detailed information on the use of antipsychotics, cholinesterase inhibitors and benzodiazepines was extracted. No papers were excluded based on their quality.

**Conclusion:**

No single guideline contains enough information on the risk, prevention, and management of medication-induced delirium to sufficiently support clinical decision making.

**Supplementary Information:**

The online version contains supplementary material available at 10.1007/s11096-024-01861-4.

## Impact statements

- No single comprehensive guideline currently provides sufficient information to guide the prescribing and management of medication induced delirium in adult patients.
- The limited understanding of medication-associated risks in the causation, treatment and prevention of delirium calls for clinical pharmacists to play a more central role in the multidisciplinary management of these patients.

## Introduction

Delirium is an acute disturbance in attention and cognition associated with significant functional decline and distress to the patient [1, 2]. The overall prevalence of delirium in older adults is low (1–2%) but increases with age, rising to 64% by the age of 85 years [3, 4]. Delirium impacts up to 30% of hospitalized patients with advanced age, cognitive decline, and medical or surgical comorbidity rising to 70% in mechanically ventilated patients [5, 6]. A recent scoping review summarized that delirium resulted in increased adjusted healthcare costs ranging from $1,532 to $22,269 per patient depending on included cost categories, country, and the type of hospital department [7, 8]. Delirium Superimposed on Dementia (DSD) occurs when a person with pre-existing dementia develops delirium, it is much more common than delirium alone [9]. Differentiating between delirium and dementia can be challenging, especially when both conditions coexist, and the clinicians do not know the patient’s baseline cognitive status. DSD is associated with higher rates of mortality and institutionalization, compared to inpatients with delirium or dementia alone [10].

Literature reports that any stressors to the baseline homeostasis are a vital cause in the development of delirium in older adults and dementia patients. These include but are not limited to substance intoxication or withdrawal, medication side effects, infection, surgery, metabolic derangements, pain, or common conditions such as constipation or urinary retention [2]. Despite the heterogeneity in causative factors, delirium is often treated as a common end point attempting to treat all instances of delirium similarly [2]. A systematic review published a comprehensive list of 33 predisposing, and 112 precipitating factors associated with delirium across all settings in a bid to improve the management and prevention success for patients [11]. From a patient safety point of view medication-associated factors are of particular relevance as they present a key strategy in the management of delirium [11]. However, no further detail is provided that would allow their correct dosing and management. Published literature provides evidence that any medication that increases the anticholinergic burden, exhibits a sedative- or antimuscarinic property; precipitates a serotonin syndrome or sleep disturbances is thought to carry a substantial and cumulative risk. In addition, age-related pharmacokinetic and pharmacodynamic changes as well as kidney and liver changes present a risk due to the effects on drug metabolism and a possible increase in blood–brain barrier permeability. Considering the unique challenges to patient safety pharmacological measures pose in the precipitation and treatment of delirium, there seems to be a paucity of comprehensive medication information to support clinical decision making.

### Aim

This study aims to review and summarise the information provided in neurology guidelines on medication-induced delirium in patients with and without dementia to inform guidance on prevention and management strategies.

## Method

### Protocol and registration

The systematic review protocol was registered with the International Prospective Register of Systematic Reviews (PROSPERO) [12] [ID: CRD42022366025] and was reported following the Preferred Reporting Items for Systematic Reviews and Meta-Analyses (PRISMA) statement [13].

### Inclusion and exclusion criteria

The Population Intervention Control Outcomes and Study Design (PICOS) criteria were used to assess study eligibility [14]. Protocols, standard procedures, guidelines, consensus guidelines, white papers, frameworks, toolkits, action plans, and strategic documents were included (referred to as “guidelines” throughout). Included were all documents detailing the prevention and management of medication-induced delirium in patients with and without dementia across all clinical settings, except for the peri-operative setting. No geographical exclusions applied (Table S1).

### Searches and study selection

A comprehensive list of 114 Neurological and Medical Societies of interest was compiled (Table S2). All websites were hand searched systematically. To complement the search, Guideline Central and PubMed database searches were included. A database search strategy was developed with the help of a research librarian at Innsbruck University (delirium [MeSH], medication therapy management [MeSH], Hallucin*, altered mental status; confusion [MeSH], Encephalopathy*, Cognitive*; Drug induced; Guidelines; Consensus) [15, 16]. Additional manual searches of related studies listed in the references, footnotes and citations were carried out to include any relevant additional guidelines. All searches were re-run prior to the final analysis on the 2nd September, 2023. Only full-text publications in English published since 2000 were included to capture any prescribing practice changes. No unpublished studies were sought (Table S3).

### Quality assessment

Title, abstract and full-text screening was carried out by two researchers (GBP/RM) independently with discrepancies resolved by discussion. A third reviewer was consulted for any unresolved discrepancies (AEW). During the screening process reviewers were blinded to the other’s decisions. Excel® (Microsoft 365) Software was used to manage the screening process. The Appraisal of Guidelines for Research and Evaluation II (AGREE II) for qualitative studies was used to conduct the quality assessment of all included papers [17]. This was performed independently by two reviewers (GBP/AEW) for all papers and any discrepancies resolved by consultation with a third (FJ). No papers were excluded based on their quality as suggested by Dixon-Woods et al. (2006) [18].

### Data extraction

A data extraction tool was designed based on the Cochrane Effective Practice and Organization of Care Review Group (EPOC) checklist [19]. The tool was piloted by three reviewers (GBP/RM/AEW) independently on two articles. All inconsistencies were resolved by discussion and the data extraction form finalized. All three assessors (AW/ASH/DD) extracted data independently.

### Data synthesis

Medication causes of delirium were extracted and a mixed approach to synthesis used (quantitative analysis by drug class combined with narrative synthesis for guideline characteristics). To minimize bias, extraction was undertaken independently by two researchers (GBP/IT) with discrepancies resolved by a third (AEW).

### Reflexivity

All authors are pharmacists. Four researchers have an extensive background in research and practice with the other two being assistant pharmacists. All researchers have experience in counseling patients and highlighting potential unwanted drug-related problems to prescribers in relation to impaired cognition and confusion.

## Results

### Guideline characteristics

#### Selection of guidelines

**Fig. 1:**
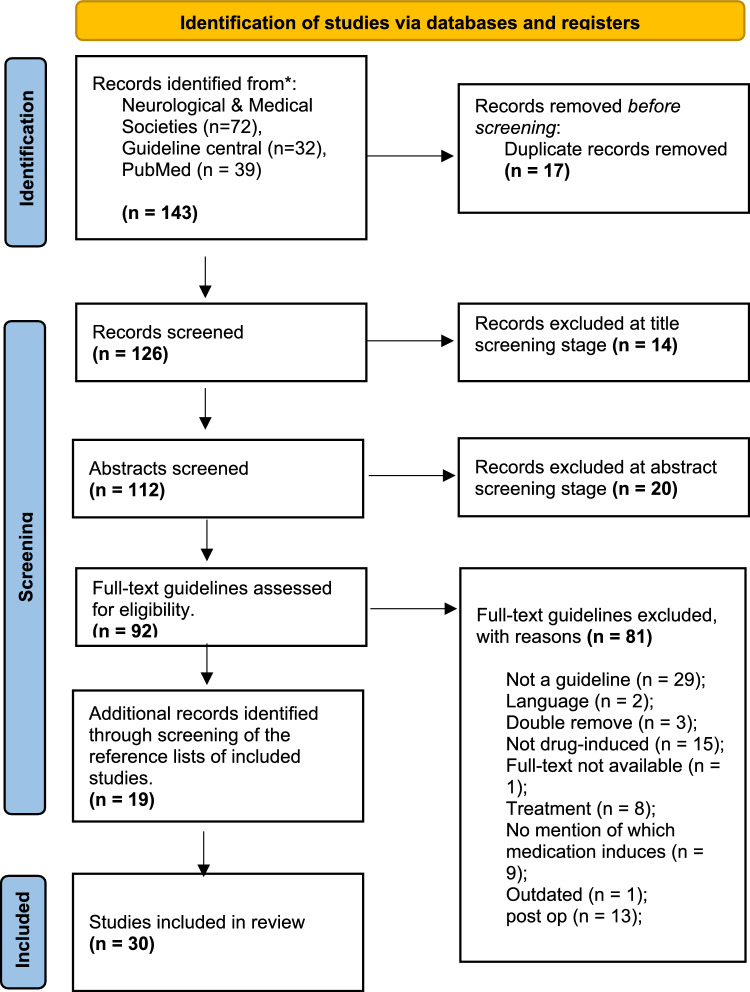
PRISMA flowchart showing the identification, screening and selection of delirium guidelines across neurological and medical professional organizations, Pubmed and Guideline Central. *Adapted from*: [13] Page MJ, McKenzie JE, Bossuyt PM, Boutron I, Hoffmann TC, Mulrow CD, et al. The PRISMA 2020 statement: an updated guideline for reporting systematic reviews. *BMJ,* 2021;372:n71. https://doi.org/10.1136/bmj.n71

The literature search yielded 143 guidelines. After screening, 30 guidelines were included (Fig. 1). Guidelines were published between 2003 and 2023 the majority of which originated from North-America (n = 11), UK (n = 8), Australia and New-Zealand (n = 5), with one paper each from Denmark, Switzerland, Iceland, Japan, India and one from a global expert group.

#### Guideline characteristics

Guidelines are either published by national and global professional organizations (n = 21; 70%) or hospitals (n = 8; 26,7%), with one published by American and Australian experts (n = 1; 3,3%). As a result, publications are applicable to a range of primary (n = 19; 63,7%) or secondary (n = 11; 36,3%) healthcare practices (Table 1).

**Table 1:** **:** Summary of guideline characteristics and quality

	Publishing Organisation	References	Year	Type of guideline	Healthcare setting	AGREE II
Canada	Registered Nurses Association of Ontario (RNAO)	[43]	2003	Guidelines	Range of Healthcare settings	***
	Canadian Coalition for Seniors’ Mental Health (CCSMH)	[51]	2006	Guidelines	Range of Healthcare settings	***
	Fraser Health	[50]	2006	Guidelines	Hospice palliative care	**
	Canadian Coalition for Seniors’ Mental Health (CCSMH)	[28]	2010	Guidelines	Not stated	***
	Registered Nurses Association of Ontario (RNAO)	[30]	2016	Guidelines	Range of Healthcare settings	***
	BC Centre for palliative care	[31]	2019	Guidelines	Hospice palliative care	***
North-Ameri**c**a						
	The American Psychiatric Association Practice (APA)	[55]	2016	Guidelines	Range of Healthcare settings	***
	International Expert opinion	[56]	2018	Guidelines	Adults in ICU	***
	Vanderbilt University Medical Center	[32]	2021	Guidelines	Adults in ICU	*
	NYU Long Island School of Medicine	[36]	2021	Algorithm	Hospital	*
	Hartford HealthCare	[33]	2022	Care Pathway	Hospital	Not assessable
UK	Intensive Care Society/ UKCPA	[23]	2006	Toolkit	Adults in ICU	***
	British Geriatrics Society / Royal College of Physicians	[27]	2006	Guidelines	Not stated	***
	The Mid Yorkshire Hospital NHS Trust	[52]	2015	Guideline	Hospital	**
	University Hospital Birmingham NHS Foundation Trust	[41]	2018	Guideline	Hospital	**
	The British Association for Psychopharmacology and the National Association of Psychiatric (BAP NAPICU)	[53]	2018	Consensus Guideline	Hospital	***
	Tayside (NHS)	[37]	2020	Guidelines	Not stated	*
	Scottish Intercollegiate Guideline Network	[44]	2019	Guidelines	Not stated	***
	National Institute of Clinical Excellence	[49]	2023	Guidelines	Hospital and long-term residential care or nursing home	***
Australia						
	Australian Health Ministers’ Advisory Council (AHMAC)	[40]	2006	Guidelines	Acute, subacute, residential care & community care settings	***
	Australian Government: Department of Health & Ageing	[26]	2010	Care Pathway	Range of healthcare settings	***
	NSW Agency for Clinical Innovation	[29]	2015	Key Principles	Hospital	**
	Australian and New Zealand Society for Geriatric Medicine	[24]	2012	Position Statement	Not stated	Not assessable
	Australian Commission of Safety & quality in healthcare	[58]	2021	Factsheet	Range of Healthcare settings	*
Denmark	Danish Health Authority	[39]	2021	Quick guide	Not stated	***
Switzerland	European Society for Medical Oncology (ESMO)	[35]	2018	Guidelines	Adults with cancer	***
Iceland	Landspitali Hospital	[34]	2015	Guidelines	Hospital	***
Japan	Japanese Society of Neurology	[54]	2017	Guidelines	Range of Healthcare settings	***
India	Indian Psychiatric Society	[45]	2018	Guidelines	Range of Healthcare settings	**
Other	Global Council on Brain Health (GCBH)	[57]	2020	Consensus Guidelines	Range of Healthcare settings	***

[High quality 70% ***, Moderate quality > 40% and < 70% **, Low quality < 40%*.] (The AGREE Research Trust, 2009)

#### Guideline quality

Most guidelines were deemed to be designed, conducted, and reported with a high quality based on the AGREE II instrument (n = 19, 63,3%) [21]. Only two guidelines could not be assessed as they are single-page care pathways or position statements [24, 33]. No guideline was excluded based on quality (Table 1**).**

### Medication at risk of inducing delirium

#### Causative medication mechanism

A summary of reported mechanisms through which medication can contribute to the development of delirium in adults is summarised in Table 2

**Table 2:** Summary of reported mechanism or risk through which medication can contribute to the development of a delirium in adults

Medication risk	References
Drug interaction	[23]
Drug withdrawal	[23, 28, 33]
Adverse effects (e.g. anticholinergic; sedative)	[24, 27–33]
The addition of three or more medications	[24, 26, 29]
Polypharmacy*	[28, 29]
Drug intoxication	[28]
Renal impairment	[26]
Drug poisoning (esp. pethidine, promethazine, illicit substances)	[23, 26]
Pharmacodynamic & Pharmacokinetic changes	[24, 29, 37]

While all guidelines clearly stated that multiple pathogenic mechanisms contribute to the development of delirium, medication is inconsistently presented as pre-disposing or precipitating factor, with no specific categorization given [22]. A variety of risks through which medication can contribute to the development of a delirium have been suggested above. Medications that cross the blood–brain barrier (BBB), increase BBB permeability (e.g. in renal failure) or have anticholinergic or dopaminergic activity are most likely to cause a delirium [23, 24]. Any medication that contributes to a serotonin syndrome such as selective serotonin reuptake inhibitors (e.g. linezolid, tramadol, and amphetamines) also increase the risk [25]. Age-related biological changes in the pharmacokinetic and pharmacodynamic properties were mentioned alongside disease- related kidney and liver impairment [26] and resultant drug toxicities [24, 27–33]

^*^No exact definition of polypharmacy is given

#### Medication at risk of causing a delirium

A total of 140 individual medication were listed across 30 guidelines. The medication classes who are cited most often (> 60% of the time) included sedatives (n = 24/80%), opioids (n = 22/73,3%), psychoactive drugs (n = 21/70%) + anti-convulsants (n = 14/46,7%), anti-cholinergic agents (n = 20/66,7%) and anti-histamines (n = 18/60%) **(**Table 3**).**

**Table 3:** Summary of all 140 individual medication reported to carry a risk of causing a delirium in adults organised by ATC Code

		Delirium			
ATC Code	Drugs at risk of causing a delirium	Risk	Treatment	Comment	References
A	Alimentary Tract and Metabolism				
***A02BA***	*H*_*2*_*-receptor antagonists*				
	Cimetidine	(✓)		Cimetidine carries the highest delirogenic potential	[23, 34, 40, 43, 49, 56]
				Overall evidence for the delirogenic potential of H2-Antagonists is inconclusive	
				Check dose adjustments in patients with renal impairment	
A03B	*Semisynthetic Derivatives*				
	Atropine	✓		Avoid due to antimuscarinic effect	[23, 35, 43]
	Belladonna alkaloids	✓		Avoid due to anticholinergic (cumulative) effect	[35]
	Butylscopolamine	✓		Avoid due to antimuscarinic effect	[23]
A03F					
	Promotility (kinetic) agents	✓		Avoid due to anticholinergic (cumulative) effect	[26, 51]
A04A					
	Prochlorperazines, Scopolamine, Metoclopramide	✓		Directly delirogenic	[20, 23, 32–35, 43]
				Anticholinergic drugs with the highest potential of causing symptoms of delirium	
				Metoclopramide should be avoided in high-risk patients	
A07AA	*Antibiotics*				
	Fludrochinolones	✓		Fludrochinolones carry the highest risk	[23, 28, 31, 34, 40, 43]
	Metronidazole; Oxazolidinone, Benzylpenicillin i.m/ Penicillin G; cephalosporins	✓		Antibiotics other than Fludrochinolones have been cautioned to cause cognitive impairment	
B	Blood and Blood forming organs				
C	Cardiovascular System				
***C01B***	*Antiarrhythmics*				
	*Digoxin;* Disopyramide; Amiodarone; Lidocain; Quinidine and Tocainide	✓		Careful monitor renal function to avoid toxicity	[23, 36, 40, 43, 49]
				Disopyramide has a high anticholinergic effect	
				Amiodarone induced sleep disturbances	
***C02A***	*Antiadrenergic Agents, Centrally Acting*				
	Clonidine, Methyldopa	(✓)		Causes changes in mental state	[49]
				Methyldopa has severe CNS side effects	
***C02C***	*Antiadrenergic Agents, Peripherally Acting*				
	Prazosin	(✓)		Causes cognitive impairment	[40, 43]
**C03C**					
	Furosemide	✓		Avoid due to anticholinergic (cumulative) effect	[49]
***C07A***	*Beta Blocking Agents*				
	Propranolol, Metoprolol, Atenolol, Elenolol, Timolol	✓		Propranolol & Metoprolol carry the highest delirogenic potential	[34]
***C08***	*Calcium Channel Blockers*				
	Dihydropyridines, Verapamil and Diltiazem	✓		Dihydropyridines have the highest delirium risk	[24, 34, 43, 52]
				Other Calcium Channel Blockers cause cognitive impairment	
D	Dermatologicals				
***D01***	*Antifungals*				
	Amphotericin B, Ketoconazole	(✓)		An indirect risk factor for delirium	[43]
G	Genito Urinary System and Sex hormones				
***G04B***					
	Oxybutinin	✓		Avoid due to anticholinergic (cumulative) effect	[28, 34, 38]
	Tolterodine	✓		Avoid due to anticholinergic (cumulative) effect	[28, 34]
H	Systemic Hormonal Preparations, Excl. Sex Hormones and Insulins				
***H02***	*Corticosteroids*				
	Hydrocortisone sodium succinate; prednisolone; prednisone; Methylprednisolone and Dexamethasone	✓		Idiosyncratic effects of corticosteroids, level of evidence is inconclusive	[16, 23, 24, 28, 31, 43, 50]
				Individual tendencies of causing sleep disturbances, anticholinergic side effects, hypothalamicpituitary-adrenal axis abnormalities	
				Avoid high doses, long-term therapy, and premature or abrupt discontinuation	
J	Anti-infectives for Systemic Use				
***J05***	*Antivirals*				
	*Ganciclovir, Acyclovir*	(✓)		An indirect risk factor for delirium	[35]
L	Antineoplastic and Immunomodulating Agents				
M	Musculo-Skeletal System				
M01A					
	Aspirin, Ibuprofen, Naproxen, Difflunisal and Sulindac	(✓)		NSAIDs can cause acute renal failure	[34–36, 40, 43]
				They pose an indirect risk factor for delirium	
Indomethacin		✓			
				They are all associated with cognitive impairment	
				Indomethacin is toxic that can induce delirium	
M03B					
	Baclofen, Orphenadrin (+ Paracetamol), Orphenadrin (+ Diclofenac), Methocarbamol, Chlorzoxazone, Carisoprodol, Cyclobenzaprine and Trihexyphenidyl	(✓)		Have all been associated with cause cognitive Impairment	[24, 32, 34, 40, 43]
N	Nervous System				
N02A	*Opioids*				
	Pethidione, Meperidine, Codeine, Hydrocodein, Propoxyohene, Hydromorphone, (Buprenorphine), Tramadol	✓		Pain treatment should be multimodal	[23, 27, 28, 31–34, 37, 43, 44, 49, 50, 52]
				If pain is not the cause of delirium opioid dose should be decreased	
				Caution: cognitive dysfunction, and hemodynamic/respiratory impairments, anticholinergic effect, opioid withdrawl and opioid induced neurotoxicty	
				Morphine & Fentanyl no significant risk	
				Avoid Transdermal fentanyl for non-cancer pain in opioid naïve patients	
N02B	*Other Analgesics and Antipyretics*				
	Nefopam	✓		Inhibits the reuptake of serotonin, dopamine, and noradrenaline	[56]
N03	*Antiepileptics*				
	Phenobarbitol, Primidone and Clonazepam, Valproic acid, Carbamazepine and Phenytoin, Mysoline	✓		Anticonvulsants are frequently associated with deteriorating cognitive impairment	[23, 28, 34, 44]
				Ongoing need and dose of anti-convulsants should be carefully considered	
	Pregabalin, Gabapentin, Lamotrigine, Topiramat and Vaplproate	N/A			
				One causative mechanism could be the impact on REM sleep	
				Mysoline is cautioned in palliative care patients	
N04					
	Bromocriptine, pergolide, amantadine, selegiline, pramipexole, ropinirole, Biperiden, Levodopa/ Carbidopa Procyclidin, Rotigotin, Benztropine	✓		High-risk medication due to their dopamine agonist and anticholinergic effects	[27, 28, 31, 34, 49]
				Levodopa should not be stopped	
	Levodopa	X			
	Rivastigmine and donepezil	✓			
N05A	*Antipsychotics*				
	Haloperidol, Quetiapine, Risperidone,	✓	✓	Risk is dose dependent; age of patient	[31, 34–43]
				Lowest effective dose Haloperidol, Quetiapine or Risperidone may be used as pharmacological treatment	
	Olanzapine, Thioridazin, Fluphenazine, Prochlorperazine, Perphenazine, Trifluperazine and Chlorpromazine, Phenothiazine	✓	X		
				Avoid in Parkinson’s disease & Palliative care	
				Caution: extrapyramidal symptoms, sedation, anticholinergic effects including increased confusion, cardiovascular effects and tardive dyskinesia	
	Lithium	✓		Poisoning in combination with other antispychotics	[35]
N05C(D)	*Hypnotics Sedatives (Benzodiazepines)*				
	Triazolam, Midazolam, Lorazepam, Alprazolam, Oxazepam, Diazepam, Clobazam and Chlordiazepoxide	✓	X	Markedly increase the odds of delirium; aggravate delirium; morbidity & mortality	[23, 29, 33–35, 37, 38, 43, 44, 46, 47]
				Risk is dose dependent	
				Should never be used as a first choice to treat insomnia, agitation or delirium	
				Do not use unless management of alcohol withdrawal, acute seizure management, palliative sedation to reduce seizure risk, myoclonus, muscle tension, acute agitation crisis or terminal delirium	
	Zolpidem	✓		No other Z-drugs have been described	[34]
	Dexmedetomidine			Superior to all other agents, including midazolam and placebo, showing a significant reduction in the incidence of agitation, confusion and delirium	[53, 70]
				Unclear if can inherently reduce delirium or merely reduce the need for delirogenic drugs	
				Is not recommended for the prevention of delirium	
N06AA	*Non-selective monamine reuptake inhibitors (Tricyclic)*				
	Amitryptiline, Imipramine, Clomipramine, Opipramol, Doxepin, Imipramine & Desipramine	✓		Antidepressants that carry the highest delirogenic potential	[27, 34, 36]
				Have a considerable anticholinergic potential	
N06AB	*Selective serotonin reuptake inhibitors*				
	Paroxetine	✓		Antidepressants that carry the highest delirogenic potential	[23, 34, 36]
	Citalopram, Escitalopram, Fluoxetine, Fluvoxamine and Sertraline	(✓)		SSRI’s and Tricyclic antidepressants both have the potential to cause REM sleep disturbances	
				Can cause electrolyte imbalances such as hyponatremia	
N06AX	*Other antidepressants*				
	Duloxetine, Milnacipran, Venlafaxin	✓		Likely to cause symptoms of serotonin syndrome	[34]
				Taper off SSRI/SNRI, don’t stop abruptly	
P	Antiparasitic Products, Insectecides and Repellents				
R	Respiratory System				
R03B					
	Glycopyrronium Bromide	✓		Avoid due to anticholinergic (cumulative) effect	[32]
R03DA	*Xanthines*				
	*Aminophylline*	✓		Anticholinergic effects and can affect sleep	[41]
R06	*Antihistamines*				
	Brompheniramine, Hydroxyzine, Promethazine	✓		First generation antihistamines carry a higher delirogenic potential compared to newer generations of antihistamines	[34, 40, 42, 43, 88]
				Carefully consider the ongoing need	
	Diphenhydramine	✓	X	Diphenhydramine in particular should not be administered	
	Loratadine, Meclazine	✓		Loratadine and Meclazine exhibit an anticholinergic potential	
S	Sensory Organs				
S01	*Opthalmologicals*				
	Homatropine	✓		Avoid due to anticholinergic (cumulative) effect	[43]
V	Various				

Steroids (n = 16/53,3%) anti-depressants (n = 12/40%), H_2_-Antagonists (n = 12/40%), anti-hypertensives (n = 11/36,7%), anti-parkinsonian (n = 11/36,7%), anti-arrhythmics (n = 10/33%), antibiotics (n = 9/30%) and non-steroidal anti-inflammatory agents (NSAIDs) (n = 8/26,7%) were also mentioned (Supplementary Table 4).

#### Sedatives

##### Antipsychotics:

Delirium caused by antipsychotics depends on the dose of the drug and the age of the patient [34]. While antipsychotics are considered a direct risk factor [35] especially in palliative care [31], low dose haloperidol, quetiapine or risperidone may be used as pharmacological treatment for hyperactive delirium where behavioral problems (e.g. severe agitation) and emotional disturbance (e.g. severe anxiety) persist [36]. In older people, caution must be observed due to their side-effect profile, including extrapyramidal symptoms, sedation, anticholinergic effects including increased confusion, cardiovascular effects, and tardive dyskinesia [37]. Antipsychotics reduce the efficacy of anticholinesterase inhibitors (AChEIs) [38] and may trigger or aggravate delirium [39–41].

Due to the potential for harm and insufficient evidence for the efficacy of antipsychotics in the prevention and treatment of delirium, these medications should be administered at the lowest effective dose and for the shortest period of time in patients who are severely agitated and/or at risk of harming themselves and/or others [42]. The use of first-generation antipsychotics can be associated with neurological side effects and QTc prolongation [40]. The use of haloperidol and risperidone for agitation/psychosis in a dosage of 0.25–4 mg/day and 0.5–2 mg/day respectively carries the following cautions: EPS symptoms (+ + +), QTc prolongation (+ + , more with IV use) and orthostatic hypotension ( +) [36]. The use of haloperidol and risperidone should be avoided when treating mild delirium in palliative patients should be avoided [31]. Olanzapine, thioridazine, fluphenazine, prochlorperazine, perphenazine, trifluoperazine and chlorpromazine were all identified as medications that carry a risk of inducing cognitive impairment and/ or delirium [36, 43].

##### Benzodiazepines:

Benzodiazepines markedly increase the odds of developing delirium in hospital/surgical wards, in residential/ community care (OR 3.0 95% CI 1.3 to 6.8) and palliative patients [37, 40, 44]. The higher the dose and the longer acting the benzodiazepine, the greater the risk [45]. Benzodiazepines should only be used for alcohol withdrawal, acute seizure management and in palliative sedation to reduce seizure risk, myoclonus, muscle tension, or acute agitation crisis [31, 44].

In addition to evidence that benzodiazepines can trigger or aggravate delirium in older adults, benzodiazepines and other sedative-hypnotics significantly increase the risk of morbidity (e.g. falls, delirium and hip fractures) and mortality [39, 46]. They should never be used as a first choice to treat insomnia, agitation, or delirium [47]. Dementia patients taking benzodiazepines [37, 45] have an increased risk of developing a delirium, falls, worsening agitation, disorientation, stroke, and premature death [47].

Short-acting benzodiazepines can cause cognitive impairment [43]. Lorazepam (medium acting) should only be used in patients with alcohol withdrawal or terminal delirium [33, 36], while alprazolam and oxazepam (medium acting) are not flagged as toxic as lorazepam [43]. Diazepam, clobazam and chlordiazepoxide (long acting) are all considered directly delirogenic drugs [23].

##### Z-substances/ hypnotics:

Zolpidem, a non-benzodiazepine Z-drug was shown to cause confusion, disorientation, and delirium [34, 48]. No other Z-drugs were described.

##### Psychoactive drugs and antimanic agents:

Most guidelines stated that haloperidol is the treatment of choice to manage delirium, however long-term haloperidol use is considered a delirium risk factor [40, 43]. The guideline from the Registered Nurses Association of Ontario (2003), states that thioridazine, fluphenazine, prochlorperazine, trifluoperazine, and perphenazine cause cognitive impairment in older patients. So do chlorpromazine, olanzapine and phenothiazine [45]. Since lithium has a narrow therapeutic window, serum lithium levels should always be monitored especially in patients with renal impairment [34, 40]. Lithium poisoning should be suspected if the patient is on combined treatment of lithium and other antipsychotics. Lithium is therefore considered an indirect risk factor for delirium [35].

#### Opioids

Pethidine [34] and meperidine [28] are the opioids most likely to precipitate a delirium while oxycodone seems least likely [34, 44]. Morphine or fentanyl were not significantly associated with delirium [44]. However transdermal fentanyl patches are contraindicated for non-cancer pain in opioid naïve patients as they pose a significant risk in the precipitation of delirium [37]. Codeine [23], dihydrocodeine [43], propoxyphene [43] and hydromorphone [28] all carry a reported delirogenic risk, with buprenorphine causing excessive drowsiness when combined with alcohol or central nervous system depressants [33]. Combining tramadol with other drugs that affect serotonin levels should be avoided [34].

#### Psychoactive drugs

##### Anti-convulsants

Phenobarbital, primidone, and clonazepam cause more cognitive impairment than valproic acid, carbamazepine, and phenytoin [34]. One causative mechanism could be the impact on rapid eye movement (REM) sleep or sleep fragmentation of carbamazepine, phenytoin, and phenobarbital [23]. Mysoline is cautioned in palliative care patients [28], while the effects of pregabalin, gabapentin, lamotrigine, topiramate, and valproate are not mentioned.

##### Anti-cholinergic and antimuscarinic medications

Most guidelines state that drugs with anticholinergic activity increase the risk of delirium in older patients. They reduce the efficacy of AChEIs while also causing sedation, cognitive impairment, delirium, and falls [36, 38].

Reported anticholinergic medication included belladonna alkaloids [35, 40, 54], homatropine [43], glycopyrrolate

[32], promotility agents [28, 45, 51], and tolterodine [28, 34, 45]. Only two antimuscarinic agents with an increased risk of precipitating a delirium were reported, atropine [23, 35, 43] and hyoscine [23].

#### Antihistamines

First-generation antihistamines such as brompheniramine, hydroxyzine, promethazine and dimenhydrinate carry a higher delirogenic potential compared to newer generations of antihistamines [34, 40, 42, 43], as they cross the blood–brain barrier more readily. Diphenhydramine, in particular should not be administered to prevent or treat delirium [55]. Loratadine and meclizine also exhibit an anticholinergic potential [43].

### Therapeutic Alternatives

Therapeutic alternatives were only provided by three publications [30, 33, 52]. Only one comprehensive best practice guideline published by the Nurses’ Association of Ontario (2016) [30] offers more comprehensive suggestions. In most cases a switch to a newer generation drug is suggested. Specific dosing recommendations are never provided (Table 4**).**

**Table 4:** Suggested alternative medication provided in patients with a high risk of delirium

Drug class	Medication	Therapeutic alternative	References
Opioid Analgesics	Fentanyl	Alternatives are Hydromorphone, Acetaminophen, or Tramadol	[33]
	Morphine	Oxycodone seems to have the least chance of causing delirium. Fentanyl can also be used	[30]
Antiemetic	Metoclopramide	Ondansetron	[33]
	Prochlorperazine	Ondansetron	[33]
Antihistamine	Diphenhydramine	Alternative for allergic Rhinitis is Loratadine	[33]
	Promethazine	Loratadine	[30, 33]
	Hydroxyzine	Loratadine	[30]
H2 receptor antagonist	Cimetidine	Ranitidine or Famotidine	[30]
	Famotidine	Alternative is PPI except with Plavix, or Pantoprazole	[33]
Antipsychotics		Use lorazepam 0.5 mg PO /IM (if available)/sublingual where antipsychotics are contraindicated	[52]
	Haloperidol	Methotrimeprazine is a more sedating alternative to haloperidol; dosing 12.5 to 25 mg SC, IV or PO Q1-2H until calming occurs	[30, 33]
Tricyclic Antidepressants		Most tricyclic antidepressants are anticholinergic and can cause delirium, for example amitriptyline (other than nortriptyline), SSRIs are less known for causing delirium symptoms, but cases have been recorded where fluoxetine and citalopram were associated with delirium	[30]

### Delirium in dementia patients

Pharmacological measures for the management of delirium in dementia patients must not be considered unless the patient’s behavior has been assessed as posing a danger to themselves or others and if non-pharmacological interventions have failed to be effective. The emphasis should be on regular rather than when required medication [41] **(**Table 5**).**

**Table 5:** Summary of drugs and cautions for medication used in delirium and dementia. Adapted from [41] University Hospital Birmingham NHS, 2015 & James C. et al., 2020)

Drug	Dose	Route	Indication	Cautions
*Typical Antipsychotics*				
Haloperidol	0.5-1 mg PRN to a max of 10 mg in 24 h. Give in 0.5 mg alliquots up to 10 mg	Oral IM	Agitation/aggression when patient is at risk of harming self or staff	Avoid in patients with Parkinson’s Disease or Lewy Body Dementia. Risk of over sedation. Use atypical antipsychotics instead
			Stress and Distress in Alzheimer’s Disease and/or delirium	
			Acute delirium when non-pharmacological treatments ineffective [licensed use]	QTc prolongation and/or ventricular arrhythmias, in addition to sudden death, have been reported with haloperidol at high doses, high plasma concentrations, in predisposed patients or with parenteral use Increased risk of stroke in people with dementia Monitor for extra-pyramidal and cardiac side-effects
*Atypical Antipsychotics*				
Quetiapine	25 mg PRN/BD	Oral	An alternative to haloperidol in patients with Parkinson’s Disease or Lewy Body Dementia	Moderate effect on QTc prolongation
	12.5 mg-25 mg daily; up to 25-100 mg daily			Increased risk of stroke in people with dementia
			Psychosis, aggression, or severe agitation/anxiety in Dementia with Lewy Bodies [unlicensed use ‘off-label’]	Monitor for sedation and postural hypotension
Risperidone	Short term (1d—6 weeks) severe/persistent anxiety/aggression: 0.25mcg BD max. of 1 mg in 24 h on alternate days	Oral	Should be used with specialist guidance for certain patients with psychosis, short-term treatment (up to 6 weeks) of persistent aggression in patients with moderate to severe Alzheimer’s dementia unresponsive to non-pharmacological interventions and when there is a risk of harm to self or others [licensed indication]	Dementia with Lewy bodies Low effect on QTc prolongation Increase risk of stroke in people with dementia Monitor for extra-pyramidal and cardiac side-effects Monitor for hypotension
			Psychosis or severe agitation/anxiety in Alzheimer’s Disease [unlicensed use ‘off-label’]	
Olanzapine	4.5 to 8.2 mg/day			Dementia is predictive of a poor response to olanzapine
*Cholinesterase inhibitors*				
Donepezil			Treatment of delirium in dementia	
Rivastigmine			Prevention of delirium in vascular dementia Visual hallucinations in LBD	
*Benzodiazepines*				
Lorazepam	0.5 – 1 mg PRN up to two hourly (maximum 3 mg in 24 h)	Oral IM or IV	2nd line for short term management of agitation e.g. if urgent scans needed	Can cause or worsen respiratory depression
			An alternative to haloperidol in patients with dementia with Lewy bodies and those with Parkinson’s disease	Benzodiazepines markedly increase the probability of delirium developing in a variety of settings
			Management of delirium in Parkinsonism or Lewy Body dementia as an alternative to quetiapine [unlicensed use ‘off-label’]	
			Short-term treatment (typically 1–2 days) of severe agitation/ anxiety in delirium [unlicensed use ‘off-label’]	
Midazolam	1.25 – 5 mg	Oral or IV	Same as other benzodiazepines	Very quick acting but short-lived

## Discussion

This systematic review provides a unique summary of the specific medication related information available to support prescribing and management of delirium patients in practice which extends beyond the advice provided in any one specific practice guideline. All 140 identified drugs across 30 drug classes span all but four ATC Codes. Overall, there is a distinct lack of specifc medication related detail on mechanism of action, dosage, route of administration, indication, cautions and therapeutic alternatives provided on medication-induced delirium in patients with and without dementia to guide safe prescribing in practice. Nervous System Drugs (ATC N) such as sedatives, opioids and psychoactive drugs carry the highest risk alongside antihistamines (ATC R) and steroids (ATC H). Medication affecting the alimentary tract (ATC A) and the cardiovascular system (ATC C) are also frequently mentioned. The advice in relation to these drug classes is mostly generic and lacks specificity which could be reflective of our general lack of understanding of the underlying pathophysiology of delirium. Challenges in the management of delirium are reflected in the advice on the use of benzodiazepines and antipsychotics. While Benzodiazepines are reported to markedly increase the odds of delirium, aggravated delirium, morbidity, and mortality, dexmedetomidine is stated as being superior to all other agents in significantly reducing the incidence of agitation, confusion, and delirium itself [44]. The lowest effective dose of haloperidol, quetiapine and risperidone are recommended for use as treatment of delirium but carry a caution of extrapyramidal and anticholinergic side effects [31]. Few alternative treatment suggestions are provided relating mainly to newer generations of drugs within drug classes. While very few documents provide specific guidance on the management of delirium in dementia patients it was possible to extract quite detailed information on the use antipsychotics (typical/ atypical), cholinesterase inhibitors and benzodiazepines including dosing instructions from one very detailed regional NHS guideline [37].

### Medication risk

The inherent lack of understanding of medication risk associated with the causation and treatment of delirium in adult patients with and without dementia is reflective of the general lack of understanding of the pathophysiology of delirium [87–89]. Considering the heterogeneity of symptoms associated with delirium, none of the guidelines specify the most common delirium symptoms associated with the different medications/ medication classes [15, 16, 66]. Merely terms such as “delirium” and “cognitive impairment” are reported, not however hallucination, confusion, disorientation, neuroleptic malignant, syndrome or encephalopathy. Therefore, it is unclear which symptom profile each individual medication contributes to. A further gap identified is the paucity of reported potentially cumulative drug combinations. While some combinations are provided e.g. orphenadrine + paracetamol or diclofenac + orphenadrine [45] there is no clear indication of which drug combinations to avoid. Australian guidelines reference a more generalized measure quoting the addition of three or more medications during inpatient stay [24]. This seems to date back to a single study from 1996 [67] which in turn, does not provide evidence for this claim. Other guidelines state the occurrence of “polypharmacy” as a contributing risk factor without a definition for polypharmacy [28, 29]. The same is true for a lack of standardized methodology to quantify the risk of individual drugs [11].

### Treatment of delirium

It is commonly accepted that pharmacotherapy should not be used as a treatment for delirium as medication could be an indirect risk factors for the development of delirium [68]. Laurentani proposes that non-pharmacological approaches should be first line with the main goal being the identification of the underlying cause [69]. Should medication treatment be needed, when behaviors pose a safety risk, or when there is a risk of interrupting essential medical care, the lowest effective dose of haloperidol, quetiapine, and risperidone should be used [42, 70]. A meta-analysis published in 2016, concluded that there is insufficient evidence to support the routine use of antipsychotics to prevent and treat delirium with a call for more rigorous well-powered randomized-controlled trials (RCTs) in high-risk populations [71]. The same is true for other strategies such as cholinesterase inhibitors, alpha-2 agonists, and melatonin receptor agonists (including melatonin itself). None show a clear benefit for the use of pharmacotherapy to prevent delirium, which may explain why these have not been included in the guidelines [72]. Orexin antagonists, suvorexant and lemborexant, while having been suggested to have a preventative effect on delirium are also not mentioned [73, 74]. Dexmedetomidine, a highly selective alpha-2 adrenoreceptor agonist, used to manage pain and sedation in the intensive care unit (ICU), is the only reported medication known to reduce the incidence of delirium and agitation in intensive care patients [75]. While the exact mechanisms for this is not fully understood, several publications seem to point towards a downregulation of the HMGB1-TLR4-NF-κB signaling pathway by activating alpha-2 adrenergic receptors and stimulation of the vagus nerve via a vagal- and alpha-7 nicotinic acetylcholine receptor-dependent mechanism [76–78].

### Delirium and dementia

Dementia is a predisposing factor for delirium [relative risk (RR):2.3–4.7] [80, 83, 84]. Cholinesterase inhibitors, commonly used in the treatment of Alzheimer’s disease and dementia have been recommended in the treatment and prevention of delirium in this patient group and critically ill patients without dementia. A recent comment by Liebermann O., et al. (2023) detailed that when older adults with preexisting dementia were stratified based on whether they were prescribed donepezil before admission, those receiving donepezil had lower rates of delirium incidence, in-hospital mortality, 90-day post-discharge mortality, lengths of stay and duration of mechanical ventilation, despite similar illness severity and comorbidities on admission [85, 86]. As with pharmacological treatment of delirium above, high-quality evidence is lacking.

### Limitations

The lack of consistent terminology in delirium research and the need to hand search the websites of all identified professional organizations may have resulted in missed publications. Limiting the search strategy for the database searches (Guideline Central and PubMed) to only include “guidelines” will not have captured other types of practice guidance. The heterogeneity of included guidelines limited the synthesis to a mixed method combining quantitative and narrative elements. Exclusion of non-English language documents may have resulted in further loss of local guidelines. Databases such as web of science, embase, cochrance library were not included in the search as they focus on original research on this topic, this may have resulted in guideline omissions. The content of original research on this topic is published in a separate systematic review.

### Future research

The results from this systematic review should form the basis for the development of clear and detailed medication related management guideline aimed at providing specific prescribing advice for the medication-related risks related to the causation, treatment and prevention of delirium, despite the identified gaps within the present neurology guidelines. The lack of our pathophysiological understanding of the causes for delirium calls for a categorization of delirium into distinct aetiological subgroups to facilitate high quality research into the effect of medication related risk in delirium within different patient groups. This would also support the development of existing and future multicomponent treatment approaches to improve patient safety. Analysis of large epidemiological and real-world databases are required to identify pertinent associations between medication, delirium, and other confounding factors.

## Conclusion

A uniquely comprehensive summary of the specific medication related information available to support prescribing and management of delirium in practice is provided. This extends beyond the advice conventionally found in any single resource. The overall paucity of detailed medication related information reflects the lack of our inherent understanding relating to the pathophysiology of delirium in different patient groups. There is an urgent need for research into risks and causes in distinct aetiological subgroups to support the development of clear and detailed holistic multicomponent approaches to delirium management.

## Supplementary Information

Below is the link to the electronic supplementary material.

Supplementary file1 (DOCX 32 KB)
