# Supplementary material for: Medication-induced causes of delirium in patients with and without dementia: a systematic review of published neurology guidelines
Source: Int J Clin Pharm. 2025 Feb 19;47(3):606–23. doi: 10.1007/s11096-024-01861-4 (PMC12125033; doi:10.1007/s11096-024-01861-4)
Supplement: Supplementary file 1 — Supplementary file1 (DOCX 32 KB) [file 11096_2024_1861_MOESM1_ESM.docx]

***Supplementary Table 1.:*** *Study inclusion criteria*

| Inclusion criteria |
| --- |
| - Protocols, standard procedures, guidelines, consenus guidelines, white papers, frameworks, action plans, strategic documents. - Documents detailing the prevention and management of medication induced delirium irrespective of the time to onset of delirium, treatment or response to treatment. - Delirium medication in the context of dementia irrespective of the type and stage of dementia or the pharmacological treatment used. - All clinical settings except for the peri-operative setting. - No geographical exclusions - Language English |

| Association of Anaesthetist of Great Britain and Ireland (AABI) |
| --- |
| American Academy of Neurology (AAN) |
| American Delirium Society (ADS) |
| American Geriatrics Society (AGS) |
| American Psychiatric Association (APA) |
| American Society of Health-System Pharmacists (ASHP) |
| Armenia International Brain Research Organization (IBRO) |
| Association of British Neurologists |
| Australasian Delirium Association |
| Australian and New Zealand Society for Geriatric Medicine (ACSQH) |
| Australian commission on Safety and quality in health care (NSGHS) |
| Australian Health Ministers' Advisory Council (AHMAC) |
| Austrian Society of Neurology |
| Basel Dementa Derlirium Program |
| Belgium Neurological Society |
| Brain Research Society (BARD) in Turkey |
| Brain Research Society of Finland (BRSF) |
| British Geriatrics Society (BGS) |
| British Neuroscience Association (BNA) |
| Bulgaria Society of Neurology |
| Bulgarian Society of Neurology |
| California Society of Health-System Pharmacist (CSHP) |
| Canadian Association for Neuroscience (CAN-ACN) |
| Canadian Coalition for Seniors´ Mental Health (CCSMH) |
| Canadian Institute of Neuroscience, Mental Health & Addiction (INMHA) |
| Canadian patient safety Institute (CPSI) |
| Canadian Society of Hospital Pharmacists (CSHP) |
| Croatian Society of Neuroscience |
| Cyprus Neurological Society |
| Czech Neurological Society |
| Danish health authority |
| Danish Society for Neuroscience |
| Dutch Association of Clinical Geriatrics |
| Dutch Association of Psychiatry |
| Estonian Ludvig Puusepp Society of Neurologist & Neurosurgeons |
| European Brain and Behaviour Society (EBBS) |
| European Delirium Association |
| European Society for Neurochemistry |
| European Society of Anaesthesiology (ESA) |
| European Society of Medical Oncology (ESMO) |
| Federation of European Neuroscience Societies (FENS) |
| Finnish Neurological Society |
| French Society of Neurology |
| Georgian Neuroscience Association |
| German Society of Neurology |
| Hellenic Society for Neuroscience |
| Hungarian Neuroscience Society |
| Icelandic Neurological Society |
| International Behavioural Neuroscience Society (IBNS) |
| International Brain Research Organization (IBRO) |
| International Group of Researchers and Clinicals (INCOG) |
| International League Against Epilepsy (ILAE) |
| International Neuroethics Society (INS) |
| International Neuroinformatics Coordinating Facility (INCF) |
| International Pharmaceutical Federation (FIP) |
| International Society for Invertebrate Neurobiology (ISIN) |
| International Society of Neuropathology (ISN) |
| International Society of Psychoneuroendocrinology (ISPNE) |
| International Union of Physiological Sciences (IUPS) |
| Irish Institute of Clinical Neuroscience |
| Israel Society for Neuroscience |
| Italian Association of NeuroImmunology |
| Italian Society for Neuroscience |
| Japanese Society of Neurology |
| Latvian Neurologist Association |
| Lithuanian Neuroscience Association |
| Luxembourg Society of Neurology |
| Macedonian Medical Association |
| Mediterranean Neuroscience Society |
| Moldovan Scientific Society of Neurology |
| Monash Health |
| Montenegrin Neurological Association |
| National Health Services (NHS) |
| National Institute for Health and Care Excellence (NICE) |
| National Neuroscience Society of Romania |
| Network for investigation of Delirium: Unifying scientist (NIDUS) |
| Neurological Research and Practice |
| Neuroscience Ireland |
| Neuroscience Society of Turkey (TUBAS) |
| Norwegian Neurological Society |
| Norwegian Neurological Society |
| Norwegian Neuroscience Society |
| Pharmaceutical Group of the European Union (PGEU) |
| Pharmaceutical Society of Australia (PSA) |
| Pharmacy Guild of Australia (PGA) |
| Polish Neurological Society |
| Polish Neuroscience Society |
| Portugese Society of Neurology |
| PsycBITE |
| Psychiatry online |
| Queensland University of Technology QUT |
| Romanian Society of Neurology |
| Royal College of Psychiatrists |
| Scientific Society of Neurologists of the Ukraine |
| Scottish Intercollegiate guideline Network (SIGN) |
| Serbian Neuroscience Society |
| Serbian Neuroscience Society |
| Slovak Society for Neuroscience |
| Slovenian Neuroscience Association |
| Slovenian Society of Neurology |
| Sociedad Española de Neurociencia |
| Sociedade Portuguesa de Neurociencias |
| Société des Neurosciences |
| Society for Neurosciences |
| Society of critical care medicine (SCCM) |
| Studies to understand Delirium in Palliative Settings (SUNDPIS) |
| Swedish Neurological Society |
| Swiss Society for Neuroscience |
| The Dana Foundation |
| The delirium guidelines development |
| The Royal College of Physicians |
| UpToDate |
| Venderbilt University (Medical centre) |
| World Health organisation (WHO) |

**Supplementary Table S2.:** Neurological and Medical professional organisations included in the online search (n=114) in addition to PubMed and Guideline Central

***Supplementary Table S3.:*** *Systematic search results*

| Date | Database | Keywords | Hits | Filter |
| --- | --- | --- | --- | --- |
| 21.08.2023 | Neurological Orginisations (n=114) | Delirium / Guideline / Dementia | 98 | N/A |
| 02.09.2023 | Guideline Central | Delirium / Guideline / Dementia | 6 | N/A |
| 02.09.2023 | PubMed | "Delirium"[Mesh] OR Delirium AND "Guidelines as Topic"[Mesh] OR "Guideline" [Publication Type] OR Guideline* AND "drug effects" [Subheading] OR "Drug-Related Side Effects and Adverse Reactions"[Mesh] OR "Drug effect*" OR "Medication effect*" OR "drug induced" | 39 | 2000-2020 |
| Total | | | **n = 143** | |

***Supplementary Table 4.:*** *Detailed summary of all remaining drug classes mentioned less than half the time across all guidelines included in this study.*

| Medication | Summary of delirium related information |
| --- | --- |
| *Anti-depressants* | Amitriptyline, imipramine, and paroxetine have been identified as the antidepressants that carry the highest delirogenic [36]. Caution should be exercised when administering MAO inhibitors at the same time as other antidepressants [34,41]. Tricyclic antidepressants and SSRI’s both have the potential to cause REM sleep disturbances [23]. SSRIs (citalopram, escitalopram, fluoxetine, fluvoxamine, and sertraline) are less known for causing delirium symptoms, but cases have been recorded where fluoxetine and citalopram were associated with delirium. SSRIs/SNRIs (duloxetine, milnacipran, venlafaxine) are likely to cause symptoms of serotonin syndrome, especially if simultaneously treated with other serotonergic drugs such as other antidepressants, tramadol, and migraine drugs [34]. Delirium induced by antipsychotics depends on the dose of the drug and the age of the patient. Care should be taken to stop the treatment of SSRI/SNRI drugs suddenly due to possible withdrawal symptoms, and therefore, it is recommended to taper off treatment slowly. SSRIs can cause electrolyte imbalances such as hyponatremia [34]. Most tricyclic antidepressants (clomipramine (NSMRI), opipramol, doxepin, imipramine & desipramine) are anticholinergic and can therefore cause delirium [27,34]. |
| *H_2_-Antagonists* | Cimetidine carries the highest delirogenic potential, according to the guidelines due to its anti-carbonic effects [34]. While the overall level of evidence for the delirogenic potential of H2-antagonists is inconclusive [56], there is a clear instruction to check dose adjustments in patients with renal impairment [40]. Nizatidine [43], ranitidine [23] and famotidine [34] all have a lower delirium risk compared to cimetidine [49]. |
| *Anti-hypertensives* | Beta-blockers, especially fat-soluble ones such as propranolol and metoprolol, carry the highest delirogenic potential [34]. Their sleep disturbing adverse effects (Insomnia, ↓ REM, nightmares) may add to the precipitating tendencies [23]. Atenolol is thought to have a direct delirogenic potential [23] and atenolol, timolol and prazosin are reported to cause cognitive impairment [40,43], while centrally acting anti-hypertensives such as clonidine are known to cause changes in mental state [34,49] dihydropyridines have an increased delirium risk with (OR 2.4) [24], while verapamil, nifedipine, and diltiazem may all cause cognitive impairment [34,43,34,52]. Methyldopa an alpha-2-anagonist and antihypertensive is well known to cause severe CNS side effects [20,34]. |
| *Diuretics* | Diuretics can potentially cause delirium by causing dehydration and electrolyte imbalance [20,34]. Oxybutynin, tolterodine and furosemide all have a high anticholinergic property and as a result oxybutynin is absolutely contraindicated in patients with delirium [49,23]. |
| *Anti-parkinsonian* | Antiparkinsonian medicines are considered high-risk medication that contribute to the development of a delirium [28] due to their dopamine agonist [28] and anticholinergic effects [27]. Bromocriptine, pergolide, amantadine and selegiline should be considered for discontinuation [34] while levodopa should not be stopped [20]. Newer dopamine agonists such as pramipexole and ropinirole carry an equal risk of causing a delirium with a particular focus on hallucinations [20]. Amantadine, biperiden, levodopa/ carbidopa procyclidine, rotigotine, amantadine, benztropine and selegiline [28] have all been mentioned, as have cholinesterase inhibitors, rivastigmine, and donepezil [31]. |
| *Antiarrhythmics* | The main delirogenic potential of antiarrhythmic drugs is associated with digitalis [40]. Careful monitoring is required in older patients as a reduced renal function may lead to digitoxin toxicity [49], while disopyramide has a high anticholinergic effect [49] and amiodarone induced sleep disturbances and nightmares [23]. Lidocaine [36], quinidine and tocainide [43] have all been associated with cognitive Impairment. |
| *Antibiotics* | The UKCPA (2006) guideline reports that increased plasma concentrations and/ or increased blood-brain barrier permeability, for example in patients with renal failure, may make patients particularly prone to the delirogenic effects of some Antibacterial (e.g. penicillin) [23]. Numerous antibiotics can possibly cause symptoms of delirium and cognitive impairment, but fluoroquinolone (ciprofloxacin, ofloxacin and norfloxacin) have accumulated the most evidence [34,43]. It is recommended to pay close attention to the dosage of fluoroquinolones in older patients and those with reduced kidney function [33,40]. Fluoroquinolones are often an underlying cause of delirium in palliative care patients [28,31].    Metronidazole [43], oxazolidinone, benzylpenicillin I.M/ penicillin G [23] and cephalosporins (cefuroxime, cephalexin and cephalothin) have also been cautioned frequently to cause cognitive impairment [43].  Antiviral agents’ ganciclovir & acyclovir have occasionally been mentioned as indirect risk factors [35] as have antifungals, amphotericin B & ketoconazole [43]. |
| *Non-Opioid Analgesics* | NSAIDs can cause acute renal failure if taken simultaneously with drugs that are excreted by the kidneys [34]. As a result, they pose an indirect risk factor for delirium [35] and medication known to cause delirium require adjustment in case of renal impairment [40]. Aspirin, ibuprofen, naproxen, diflunisal and sulindac are all associated with cognitive impairment [43] while indomethacin is a potential toxic medication that can induce delirium [36].  Dexmedetomidine, a highly selective α2-adrenergic receptor agonist is superior to midazolam and placebo, in showing a significant reduction in agitation, confusion, and delirium [53]. It remains unclear if dexmedetomidine can inherently reduce delirium or merely reduce the need for delirogenic drugs. Since there are potential physiological concerns relating to the widespread adoption of dexmedetomidine, in addition to cost implications, dexmedetomidine is not recommended for the prevention of delirium [44]. |
| *Other*  *Muscle relaxants & Anti-spasmodics (GI)* | Gastrointestinal antispasmodics and skeletal muscle relaxants carry a significant anticholinergic effect. [24,34,40]. baclofen, orphenadrine (+paracetamol), orphenadrine (+diclofenac), methocarbamol, chlorzoxazone, carisoprodol, cyclobenzaprine and trihexyphenidyl have all been associated with cause cognitive Impairment [32,43]. |
| *Respiratory* | Drugs for asthma have anticholinergic effects and can affect sleep [23], aminophylline [41] and nefopam [56] can be associated with delirium. |
| *Anti-emetics* | Antinauseant medications were reported as a risk of causing delirium as they mostly have anticholinergic effects. Prochlorperazines [23,33,34,43] a directly delirogenic drug and scopolamine [34,35] are anticholinergic drugs and with the highest potential of causing symptoms of delirium [20]. The use of metoclopramide should also be limited or avoided in high-risk patients [32,43]. |
| Chemotherapy | The European Society for Medical Oncology [35] guideline published in 2018 contained the most information about particular chemotherapy medications at risk of inducing delirium. They report that chemotherapy medication toxicity is a direct risk factor for delirium. Together with the ESMO publication, the Fraser Health Authority (FHA) guideline from 2006 identifies specific individual chemotherapy medications because the guideline provides recommendations for individuals suffering from advanced life-threatening illnesses and experiencing delirium [35,43,50]. |
